# Substrate and standard evaluation for correlative elemental mapping of biological samples by X-ray fluorescence microscopy and laser ablation ICP-MS

**DOI:** 10.1039/d5ja00371g

**Published:** 2026-01-16

**Authors:** David Z. Zee, Soo Hyun Ahn, Andrew M. Crawford, Niharika Sinha, Qiaoling Jin, Chris Jacobsen, Evan Maxey, Barry Lai, Keith W. MacRenaris, Thomas V. O'Halloran

**Affiliations:** a Elemental Health Institute, Department of Microbiology, Genetics, and Immunology, Michigan State University East Lansing Michigan 48824 USA macrenar@msu.edu ohallor8@msu.edu; b Department of Chemistry, Michigan State University East Lansing Michigan 48824 USA; c X-ray Science Division, Advanced Photon Source, Argonne National Laboratory Lemont Illinois 60439 USA; d Department of Physics and Astronomy, Northwestern University Evanston Illinois 60208 USA

## Abstract

Analytical techniques that offer accurate, sensitive, and high-resolution elemental mapping have significantly advanced our understanding of the role of inorganic chemistry in vital biological processes. Among these, synchrotron-based X-ray fluorescence microscopy (XFM) is a particularly powerful tool for providing reliable, non-destructive quantitation of endogenous elements in biological specimens. However, its broader application is constrained by limited beamtime availability. Recent advancements in laboratory-based imaging techniques—such as laser ablation inductively coupled plasma time-of-flight mass spectrometry (LA-ICP-TOF-MS)—have significantly increased the availability and throughput of elemental mapping. Yet, quantitation with LA-ICP-TOF-MS is susceptible to matrix effects, making correlative mapping with XFM critical for validation. This presents a challenge: the two techniques require different sample preparations. LA-ICP-TOF-MS uses glass slides, while XFM requires thin, low-scatter substrates to minimize X-ray background signals. To address this, we evaluated twelve commercially available substrates previously reported for XFM to determine their suitability for LA-ICP-TOF-MS. Our goal was to identify substrates that (1) exhibit low elemental background and minimal interference with quantifying endogenous inorganic species, and (2) are compatible with both imaging modalities. As an initial step, we compared adjacent brain sections prepared on Ultralene (for XFM) and glass (for LA-ICP-TOF-MS) to establish a baseline correlative approach. Building on this, Ultralene, followed by Kapton film, emerged as the most promising candidates for enabling dual XFM and LA-ICP-TOF-MS workflows, offering low background, reliable XFM performance, and demonstrating robust elemental mapping in LA-ICP-TOF-MS. These findings support more accurate and accessible correlative imaging workflows for elemental mapping of biological samples with both modalities.

## Abstract 2

Analytical techniques that offer accurate, sensitive, and high-resolution elemental mapping have significantly advanced our understanding of the role of inorganic chemistry in vital biological processes.

## Graphical abstract

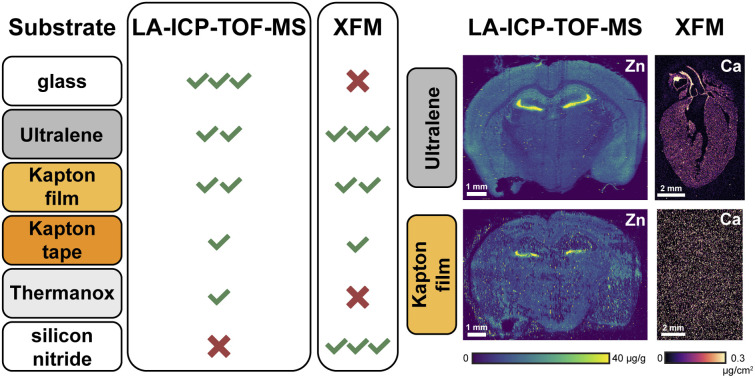

## Introduction

Accurate quantitation of fluctuations in the cellular metallome is essential for elucidating the trafficking, storage, and homeostasis of inorganic elements that support critical physiological functions.^1–5^ Dysregulated metallome fluxes are implicated in numerous diseases. For example, deficiencies in elements such as iron, zinc, or copper are associated with well-characterized pathologies, including anemia and neurodegenerative disorders, and in severe cases, conditions such as Menkes and Wilson diseases.^6–8^ While bulk elemental analysis has yielded valuable insights into inorganic physiology, it does not account for the cellular heterogeneity within tissues, where diverse cell types work together to maintain organ function. Therefore, to fully understand the role of metal homeostasis in health and disease, high-resolution spatial elemental mapping of tissue is necessary. When integrated with complementary imaging techniques, such as immunohistochemistry, elemental mapping can uncover correlations between inorganic physiology and specific cell types,^9^ offering deeper insights into disease mechanisms and the essential roles of cellular metal content in biological processes.

Investigations into the metallome at both the tissue and cellular levels have been enabled by high-resolution imaging modalities,^5,10–21^ such as synchrotron-based X-ray fluorescence microscopy (XFM),^22–24^ nanoscale secondary ion mass spectrometry,^25,26^ scanning transmission electron microscopy,^27,28^ and laser ablation inductively coupled plasma mass spectrometry (LA-ICP-MS).^29–33^ The availability of these tools has led to a rapid increase in studies probing the elemental landscapes of biological samples. For many biologists, the limited accessibility of XFM has prompted the adoption of LA-ICP-MS as a practical alternative. However, its ablative sampling process introduces elemental fractionation and more specifically matrix effects that can compromise quantitative accuracy.^34^ Matrix effects arise from tissue composition differences that influence ablation efficiency and downstream aerosol ionization. Even with mass calibration, these artifacts may skew apparent elemental concentrations. In contrast, synchrotron-based XFM—especially of thin sections (5–20 µm)—is largely non-destructive and free of matrix effects, as thin sections reduce X-ray self-absorption and biological matrices contain very low levels of heavy elements.^24^ As a result, XFM can provide more accurate quantitation of endogenous elemental content and despite its limited availability, can serve a critical role in validating LA-ICP-MS data as well as identifying matrix effects inherent to laser ablation for quantitative elemental mapping of biological samples.

A key challenge in correlative, multimodal elemental mapping is identifying a substrate compatible with both XFM and LA-ICP-MS. An ideal substrate should (1) have low background levels of the elements of interest, (2) support tissue mounting, (3) permit transfer between imaging modalities, and (4) not interfere with detection in either modality. For XFM, substrates must have minimal X-ray scattering characteristics, thus excluding glass microscope slides typically used in LA-ICP-MS.^35–38^ Substrates used in XFM include silicon nitride,^39–44^ Thermanox coverslips,^24,45–48^ Kapton tape,^48,49^ and thin polymer films developed for X-ray fluorescence spectroscopy, such as Ultralene,^50–55^ Kapton film,^56,57^ polypropylene,^56^ Etnom,^58^ and Mylar.^24,59,60^ On the other hand, thin film silicon nitride windows commonly used as substrates in XFM studies can be sensitive to rapid local heating by tens of thousands of laser pulses required to map a single sample in LA-ICP-MS experiments. Combined XFM and LA-ICP-MS mapping thus requires materials that are compatible with both the X-ray beam and the incident laser providing a means for correlative, quantitative elemental mapping. This will not only allow the study of matrix effects due to mass fractionation in LA but could also enable other correlative multimodal imaging including laser-induced breakdown spectroscopy, infrared spectroscopy, Raman spectroscopy, and various histochemical techniques.^61^

Herein, we evaluate twelve substrates (Kapton tape, silicon nitride, Thermanox microscope coverslips, Zythene, Etnom, Ultra-polyester, Mylar, Ultralene, Prolene, polypropylene, and Kapton thin films made by two manufacturers) to identify candidates suitable for both XFM and LA-ICP-TOF-MS mapping. We characterized their bulk elemental content, minimum detection limits, and impacts on quantitation by analyzing various biological tissues (liver, heart, kidney, and brain) and cryo-sectioned porcine gelatin standards. Elemental maps from both XFM and LA-ICP-TOF-MS were also compared across substrates. As an initial step toward multimodal workflows, we established a baseline correlative approach by comparing adjacent brain sections prepared on Ultralene for XFM and on glass for LA-ICP-TOF-MS; we then demonstrated that Ultralene itself supports robust elemental mapping with both modalities. Based on these assessments, Ultralene, followed by Kapton film, emerged as the most promising substrates for enabling correlative elemental imaging workflows. Nonetheless, substrate-dependent differences highlight the need for careful substrate selection to ensure accurate, quantitative, and reproducible results.

## Experimental

### Materials

Twelve substrates were purchased from manufacturers: Thermanox microscope coverslips, Kapton tape, silicon nitride membranes, Zythene, Etnom, Ultra-polyester, Mylar, Ultralene, Prolene, polypropylene, and Kapton thin films from two suppliers (for additional details of each substrate, see Table SI). For bulk analysis, all substrates were digested using sub-boiling point distilled (CEM, Matthews, NC, USA) trace metal grade HNO_3_ (70%, Fisher Chemical, Cat# A509-P212), trace metal grade HCl (37%, Fisher Chemical, Cat# A508-P500), ultrapure H_2_O (18.2 MΩ cm at 25 °C), and pre-washed (3× in ultrapure H_2_O and air dried) metal-free polypropylene conical tubes (15 and 50 mL, Labcon, Petaluma, CA, USA).

#### Analysis of bulk elemental content in substrates

Quantitation of Mg, P, S, Ca, V, Cr, Mn, Fe, Co, Ni, Cu, Zn, Se, and Mo was accomplished using inductively coupled plasma-mass spectrometry (ICP-MS) of acid digested samples. Specifically, substrate samples (ranging from 8–160 mg) were each digested in a 110 mL fluoropolymer vessel (iPrep, CEM, Matthews, NC, USA) with 5 mL of HNO_3_ and 1 mL of HCl and microwave-heated using the following program: 30 min ramp to 260 °C, held at 260 °C for 20 min, exhausted for 60 min (Mars6, CEM, Matthews, NC,USA). A 240 µL aliquot of each digestate was diluted with ultrapure H_2_O to produce a final solution of 4.0% (v/v) HNO_3_ and 0.8% HCl in a total sample volume of 5 mL. These completed ICP-MS samples were then analyzed using an Agilent 8900 Triple Quadrupole ICP-MS (Agilent, Santa Clara, CA, USA) equipped with the Agilent SPS 4 Autosampler, integrated sample introduction system (ISIS), x-lens, and MicroMist nebulizer. The elemental content of the method blanks (*i.e.*, microwave digests of acid alone) were subtracted from the elemental content of substrate digests.

Daily tuning of the ICP-MS instrument was accomplished using manufacturer supplied tuning solution containing Li, Co, Y, Ce, and Tl. Global tune optimization was based on optimizing intensities for ^7^Li, ^89^Y, and ^205^Tl while minimizing oxides (^140^Ce^16^O^+^/^140^Ce^+^ < 1.5%) and doubly charged species (^140^Ce^++^/^140^Ce^+^ < 2%). Following global instrument tuning, gas mode tuning was accomplished using the same manufacturer supplied tuning solution in kinetic energy discrimination (KED) mode (using 99.999% He, Airgas) and O_2_ mode (using 99.999% O_2_, Airgas). ICP-MS standards were prepared from a stock solution of IV-Stock-4 multi-element standard (IV-74434, Inorganic Ventures), P and S single element standards (Inorganic Ventures, Christiansburg, VA, USA) that were diluted with 4% (v/v) HNO_3_ and 0.4% (v/v) HCl in ultrapure water to produce eight serially diluted (1 : 2) standards from 50 to 0.39 ng g^−1^ and a blank (0 ng g^−1^). Internal standardization was accomplished inline using the ISIS valve and a 200 ng g^−1^ internal standard solution in 3% (v/v) HNO_3_ in ultrapure water consisting of Bi, In, ^6^Li, Sc, Tb, and Y (IV-ICPMS-71D, Inorganic Ventures, Christiansburg, VA, USA). The isotopes selected for analysis in He mode were ^24^Mg, ^44^Ca, ^51^V, ^52^Cr, ^55^Mn, ^56^Fe and ^57^Fe, ^59^Co, ^60^Ni, ^63^Cu and ^65^Cu, ^64^Zn and ^66^Zn, ^95^Mo, and ^45^Sc, ^89^Y, ^115^In, ^209^Bi for internal standardization. The isotopes selected for analysis in O_2_ mode were ^31^P^16^O, ^32^S^16^O, ^78^Se^16^O and for internal standardization ^45^Sc^16^O and ^89^Y^16^O. Continuing calibration blanks were run every ten samples, and a continuing calibration verification standard was analyzed at the end of every run for a 90–110% recovery.

#### Animal care and welfare

Animal handling protocols complied with National Research Council's animal care and welfare guidelines,^62^ and were approved by the Institutional Animal Care and Use Committee at Michigan State University. All animals were maintained on a 12 h : 12 h light cycle with standard rodent chow and water available ad libitum. Animals were anaesthetized with isoflurane and humanely euthanized by cervical dislocation.

### Synchrotron X-ray fluorescence microscopy (XFM) of substrates and tissue samples

#### Tissue sample preparation

Liver and heart tissues from mice on BALB/c background strain were excised and placed in plastic molds (Fisherbrand disposable base molds, Fisher Scientific, Waltham, MA, USA). After excision from the animal, organs were immediately embedded in optimum cutting temperature compound (Tissue-Tek OCT, Sakura Finetek, Torrance, CA, USA) and frozen in isopentane (Reagent plus grade, Sigma Aldrich, St. Louis, MO, USA) chilled with liquid nitrogen. All samples were stored at −80 °C until retrieved for sectioning using a Leica CM3050S cryostat (Leica Biosystems, Deer Park, IL, USA) with a chamber temperature of −22 °C and objective temperature of −20 °C. Samples were sectioned and mounted onto glass slides or substrates at 20 µm thickness and stored at −20 °C until transport for XFM analysis. Only a subset of the twelve screened substrates was used for tissue imaging. All substrates were first evaluated for bulk elemental content and XFM detection performance, and only those with low backgrounds, routine use in XFM cryosection workflows, and sufficient mechanical robustness were carried forward. Ultralene, Kapton film, Kapton tape, and Thermanox met these criteria. Other substrates (*e.g.*, silicon nitride and additional polymer films) either showed higher backgrounds, inconsistent detection performance, or were impractical for mounting and transferring 20 µm cryosections, and were therefore not used for XFM or LA-ICP-TOF-MS tissue mapping. Samples were transported in Styrofoam coolers in dry ice and immediately stored at −20 °C until XFM imaging was performed.

#### XFM data acquisition

Using beamline 8-BM-B of the Advanced Photon Source (Argonne National Laboratory, Lemont IL, United States), X-ray fluorescence spectra of the substrates were collected in fly scan mode at 100 µm step sizes and 50 ms dwell times with incident energy of 10 keV selected using a dual multilayer monochromator. Substrates were oriented at 45° to the incident X-ray beam, with the detector positioned at 90° to the incident X-ray beam to minimize the intensity of elastic scattered X-rays. The beam spot was focused to a full-width at half-maximum of 20 µm using a pair of Kirkpatrick–Baez mirrors. Fluorescence was measured using a Vortex 4-element silicon drift detector (SII NanoTechnology USA, Northridge, CA) running Digital X-ray Processor electronics (X-ray Instrumentation Associates) and normalized to a nitrogen-filled upstream ion chamber attached to a Stanford Research SR570 low noise current to voltage preamplifier.

For mouse tissue samples, the substrates with tissue sections were removed from the glass slides and mounted by taping all four sides of the film onto the custom-designed mount for 8BM-B beamline at the APS. Each section was raster scanned at an incident energy of 10–11 keV with a 20 µm × 20 µm beam, at a step size of 25 µm × 25 µm, and dwell time of 50 ms per pixel. Spectra were collected using a Vortex ME4 4-element silicon drift detector. Calibration of elemental intensities was performed using AXO 10× thin film AXO standards (Applied X-ray Optics, Dresden, GmbH, Germany).

Additional XFM data of murine brain tissue coronal sections were collected at the 7–2 beamline at the Stanford Synchrotron Radiation Lightsource (SSRL) with the support of Clinton Kidman. The incident energy was 13.45 keV using a Si (111) double-crystal monochromator. Samples were mounted at 45° to the incident X-ray beam, and spectra were collected using a seven-element Hitachi Vortex detector positioned at 90° to the incident X-ray beam, paired with a Quantum Detectors Xspress3 multi-channel analyzer system. The beam was focused to approximately 35 µm using an ellipsoidal polycapillary optic (Sigray, Benicia, CA, US). Samples were raster scanned at a dwell time of 300 ms per pixel and a step size of 30 µm horizontally and vertically. Mass calibration was performed by comparison to reference spectra from Micromatter standards (Mn, 47.1 µg cm^−2^; Fe, 56 µg cm^−2^; Cu, 39.4 µg cm^−2^; ZnTe, 45.8 µg cm^−2^) deposited on Mylar film. Standards were measured under the same beamline configuration at a step size of 150 µm × 150 µm and a dwell time of 300 ms to minimize contamination risk and maximize signal quality.

#### Spectral fitting

XFM data were fitted using the M-BLANK^63^ software package for spectral deconvolution. Briefly, fluorescence emissions were modelled as modified Gaussian distributions as previously described^64,65^ with a slight correction.^66^ Branching ratios,^67,68^ fluorescence yields, and emission energies were referenced using the xraylib^69^ program and the CXRO X-ray Data Booklet.^70^ Branching ratios for M-BLANK were empirically optimized^68^ during parameter creation. Once calculated, peak shapes were constrained and used in linear least squares fitting of the spectral data. Fitted fluorescence values were converted to areal concentration in µg cm^−2^ by comparison to the AXO standard (Ca, 28.87 µg cm^−2^; Fe, 3.758 µg cm^−2^; Cu, 2.034 µg cm^−2^).

#### Determination of XFM minimum detection limits

After fitting and quantitation against the AXO standard, XFM afforded elemental distributions of 600–2400 pixels for each element and each substrate. The minimum detection limit (MDL) while using a given substrate is calculated as 3*σ*. In XFM, substrate- and scatter-derived backgrounds are removed during spectral fitting, yielding background-subtracted values centered at zero; therefore, *σ* represents the standard deviation of the residual background, and 3*σ* reflects the minimum net signal distinguishable from normal background fluctuations.^71,72^

### Laser ablation inductively coupled plasma time-of-flight mass spectrometry of substrates and tissue samples

#### Tissue sample preparation

Kidneys and brains from mice on Balb/c background strain were excised and placed in plastic molds and immediately embedded in optimum cutting temperature compound. Kidney was frozen in isopentane chilled with liquid nitrogen; care was taken to avoid direct contact between the isopentane and tissue, as that caused the tissues to crack. Brain was put directly into −80 °C freezer to be slowly frozen overnight. All samples were stored at −80 °C until retrieved for sectioning using a Leica CM3050S cryostat with a chamber temperature of −22 °C and objective temperature of −20 °C. Tissue sections of 20 µm thickness were cut and mounted onto charged glass microscope slides (SuperFrost Plus, Fisher Scientific, Waltham, MA, USA) or onto Kapton tape, Thermanox, Kapton film, or Ultralene. For Kapton tape, the tissue samples were placed on the side without silicone adhesive.

Twenty-micrometer sections were selected to balance compatibility between XFM and LA-ICP-TOF-MS: increasing section thickness improves XFM areal elemental signal and reduces required beamtime, while remaining thin enough for complete ablation with the 266 nm laser. Under the optimized laser fluence used in this study (1.5–3 J cm^−2^, see below), all sections were fully ablated in a single raster pass, with no visible residual material remaining during post-ablation inspection.

Brightfield images for tissue sections were obtained with a Zeiss AxioScan 7 Microscope Slide Scanner (Carl Zeiss AG, Oberkochen, Germany).

#### Preparation and quantitation of gelatin standards

Two sets of 10% (w/v) porcine gelatin (Bloom 300, Sigma Aldrich, St. Louis, MO, USA) standards were prepared to calibrate elemental signals of LA-ICP-TOF-MS into concentrations. The first set of standards was prepared at concentrations of 0, 10, and 50 µg g^−1^ using IV-Stock-4 to calibrate concentrations of first row transition elements and the alkaline earth metals Mg and Ca. An intermediate 25 µg g^−1^ standard was originally included in the study design but was omitted due to a preparation error that resulted in inconsistent quantitative behavior. A second set of standards for Na and P was prepared at concentrations of 0, 1000, 2000, and 4000 µg g^−1^ using monosodium phosphate (Reagent Plus grade, ≥99.0%, Millipore Sigma). All gelatin standard samples were prepared using 15 mL metal-free conical tubes and maintained at 55 °C. A 10% gelatin prepared without any added standards was used to calibrate the 0 µg g^−1^ data point. A 400 µL aliquot of gelatin solution was pipetted directly onto the cryostat mounting chuck, left to freeze at −20 °C for 4 min, and sectioned at 20 µm thickness onto either charged glass microscope slides or onto a substrate: Kapton film, Ultralene, Kapton tape, and Thermanox. Each substrate was wiped with 70% ethanol before collecting the gelatin sections.

The elemental concentrations of the gelatin standards were confirmed with ICP-MS. A 100 µL aliquot of each standard was aliquoted into a pre-weighed metal-free tube and digested with 300 µL of nitric acid at 70 °C for 4 h. Following digestion, 9700 µL of ultrapure water was added to each sample. All samples were reweighed, then analyzed with ICP-MS as described above.

A separate set of gelatin standards (0, 12.5, 25, and 50 µg g^−1^) was prepared and analyzed independently for the purpose of characterizing calibration linearity (Fig. 6b); these standards were not used for quantifying tissue samples.

#### LA-ICP-TOF-MS acquisition and analysis

Tissue samples mounted on glass or on substrates described below were ablated using an ImageBIO266 laser ablation system (Elemental Scientific Lasers, Bozeman, MT, USA), which is equipped with a 266 nm laser, an ultra-fast, low dispersion TwoVol3 ablation chamber, and a dual concentric injector. The aerosolized sample was transferred to the Tofwerk icpTOF S2 mass spectrometer (TOFWERK AG, Thune, Switzerland), where the elemental content was analysed in real time according to mass/charge (*m*/*z*) ratio. Daily tuning was performed using the NIST SRM612 glass certified reference material (National Institute for Standards and Technology). High intensities for ^140^Ce and ^55^Mn were used to optimize torch alignment, lens voltages, and nebulizer gas flow while maintaining low oxide formation based on the ^232^Th^16^O^+^/^232^Th^+^ ratio (less than 0.5%). Acquisition parameters are provided in Table S2 (see SI). Laser scanning was performed in continuous scan mode with a laser repetition rate of 100 Hz, circular spots with a diameter of 10 µm, step size of 10 µm, yielding a scanning speed of 1.0 mm s^−1^ with no spot overlap in either direction. The laser fluence was set following laser power tests of each tissue on the various substrates to determine optimum laser energy for ablation (ranging from 1.5 to 3 J cm^−2^). The TwoVol3 cell provided rapid washout (*ca.* 5 ms under our operating conditions), ensuring complete signal decay between successive 100 Hz shots and allowing the icpTOF S2 to record each pulse as an independent voxel without signal mixing. Ten lines of gelatin standards were ablated using the same parameters but with the raster spacing set at 60 µm to ensure clean ablation of each raster. Each calibration line scan consisted of 320–421 pixels at a 10 µm step size (3.2–4.2 mm per line), providing several millimeters of material sampled at micrometer resolution. Elemental intensities were highly uniform across these line scans, particularly for gelatin standards on glass, confirming homogeneity at the 10 µm scale and consistent with the expected microscale uniformity of molten, spike-dissolved gelatin standards.

Scanning data was recorded using TofPilot (version 1.3.4.0, TOFWERK AG) and saved in the open-source Hierarchical Data Format 5 file format. LA-ICP-TOF-MS data were fitted using AutoSpect, a software package developed in-house that performs time-dependent drift corrections, background correction, and peak deconvolution.^73^ Image generation and concentration calibration were conducted using the Iolite software package (version 4.8.6, Elemental Scientific Lasers).^74,75^

## Results and discussion

### Substrates commonly contain µg g^−1^ levels of P, Ca, and Mn

Ideal substrates for biomedical imaging should be free of elements of interest to avoid confounding signals from biological samples, therefore knowing the elemental content of each substrate is desired. Each substrate is marketed under a trade name, and information about their exact composition varies (Fig. S1). For example, Kapton is a family of polyimide films produced by DuPont, with Kapton HN known to be poly(4,4′-oxydiphenylene-pyromellitimide).^76^ The Kapton tape tested here is explicitly labelled as Kapton HN,^77^ whereas the thin polyimide films used for XRF are marketed as “Kapton” by suppliers such as Chemplex and SPEX, but without disclosure of their exact formulations or sourcing.^78,79^ Based on reported densities and structural formulae,^78^ we infer Prolene to be polypropylene, Mylar and Ultra-polyester to be variants of polyethylene terephthalate, and Etnom to likely be polyethylene naphthalate. Thermanox is described as a polyolefin,^80^ and Ultralene shows spectral characteristics consistent with polyolefins.^50,53^ The composition of Zythene is not disclosed.

Therefore, we analysed the elemental content of commonly used XFM substrates (Table S3). When digested with HNO_3_ alone, digestates of Mylar, Ultra-Polyester, and Kapton films formed crystalline precipitates upon cooling, consistent with previous reports that complete digestion of polyethylene terephthalate requires both HNO_3_ and HCl.^81^

While the polymers consist primarily of C, H, O, and N, ICP-MS revealed µg g^−1^ levels of P, Ca, and Mn in many substrates (Table S3). Calcium was most prevalent: Mylar and Ultra-polyester contained over 2000 µg g^−1^ of Ca, while Ultralene, Etnom, and Zythene had 100–300 µg g^−1^ and the two Kapton films contained 300–550 µg g^−1^ of Ca (Table S3). Phosphorous was also common, detected at 1130 µg g^−1^ in Mylar, approximately 300 µg g^−1^ in both Kapton films, and 43 µg g^−1^ in Thermanox (Table S3). The presence of P and Ca in the Kapton films is consistent with the use of CaCO_3_ and phosphorus-containing compounds as anti-slip additives in Kapton HN.^82,83^ Among biologically relevant transition elements, Mn was found in Thermanox, Zythene, and Etnom (22–60 µg g^−1^), and Co was detected in Thermanox (0.7 µg g^−1^, see Table S3). Magnesium was also present in Thermanox (67 µg g^−1^). All remaining elements were found at levels statistically indistinguishable from the method blanks.

Substrates with lowest gravimetric concentrations of elements, in ascending order, were: Prolene and polypropylene (tied), Thermanox, Ultralene, Zythene, Etnom, the Kapton thin films, Ultra-polyester, and lastly Mylar. These results underscore the importance of quantifying background elemental content when selecting substrates for XFM or LA-ICP-TOF-MS. Notably, although Thermanox ranked third-lowest in total elemental content, it is approximately 100 times thicker than most other substrates (Table S1), which can present challenges for XFM in the form of increased diffuse scatter background.

### Experimental XFM minimum detection limits with various substrates

**Fig. 1:**
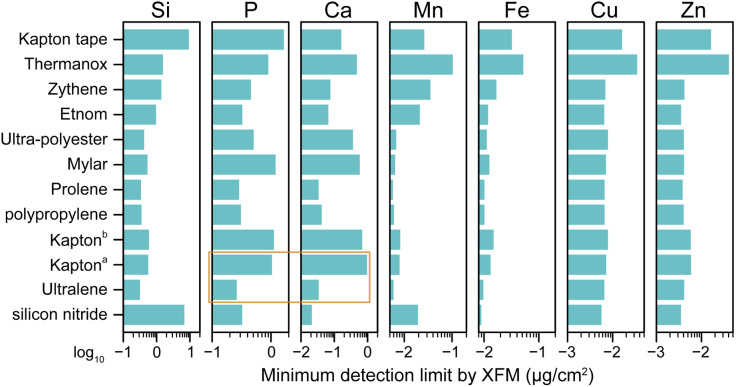
Minimum detection limits (µg cm^−2^) of Si, P, Ca, Mn, Fe, Cu, and Zn on various substrates with X-ray fluorescence microscopy (8-BM beamline, Advanced Photon Source). The minimum detection limit is defined as 3*σ*, where *σ* is the standard deviation of elemental content across all pixels in the substrate. ^*a*^Kapton film manufactured by SPEX. ^*b*^Kapton film manufactured by Chemplex. Detection limits for these elements, along with S, Cl, K, Ti, V, Cr, Co, and Ni, are tabulated in Table S4 in the SI. The orange box highlights the higher detection limits (*i.e.*, worse sensitivity) for P and Ca in Kapton film *versus* in Ultralene, which leads to poorer XFM image quality for Ca in biological samples when Kapton film is used as the substrate (see Fig. 3).

To determine the suitability of various materials as XFM substrates for biological samples, X-ray fluorescence maps of each substrate were acquired. Specifically, we determined the minimum detection limit (MDL)—the smallest areal concentration of an element that can be reliably detected in a biological specimen mounted on a substrate.^71,72^ The MDLs were evaluated for Si, P, S, Cl, K, Ca, Ti, V, Cr, Mn, Fe, Co, Ni, Cu, and Zn across twelve substrates (Fig. 1, see Table S4 for tabulated MDLs for all fifteen elements), where the MDL is defined as 3*σ*, with *σ* being the standard deviation of elemental content across all pixels of the substrate.

Each substrate's elemental MDLs in XFM (Fig. 1 and Table S4) largely correlate with its bulk gravimetric elemental content (Table S3), with some exceptions. Because X-rays are deeply penetrating, fluorescence signals arise from the full thickness of the substrate, and are accompanied by persistent inelastic scattering, which can be approximated as a combination of an exponential tail and a broadened Gaussian.^63^ Additionally, inefficient electron capture in current state-of-the-art X-ray detectors introduces low energy tailing,^64–66^ such that high-energy emissions contribute noise to lower-energy channels. Thus, the MDL of a given element in a substrate depends not only on that element's own abundance, but also on contributions from inelastic scatter and low-energy tailing caused by higher-energy emissions.

**Fig. 2:**
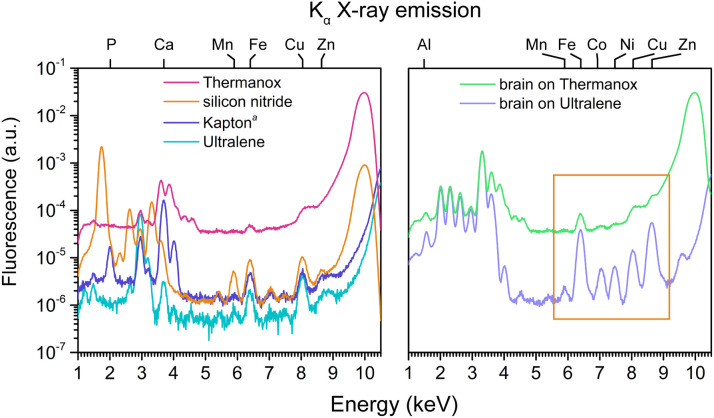
X-ray fluorescence spectra of four substrates (left panel) and spectra of brain tissue on Ultralene and Thermanox (right panel). Substrates and tissues were scanned at the 8-BM beamline at Argonne National Laboratory. Ultralene and Kapton exhibit overall fluorescence intensities comparable or lower than that of silicon nitride, while Thermanox displays *ca*. 100-fold higher fluorescence due to pronounced ineleastic scatter. This elevated background in Thermanox results in lower signal-to-noise (*i.e.*, poorer MDLs), as evidenced by the weakly discernible transition metal signals of brain tissue on Thermanox *versus* the same tissue on Ultralene (orange box, right panel). Substrate spectra shown here are representative of those used to calculate the MDLs in Fig. 1. Dwell time = 50 ms per pixel. ^*a*^Kapton film produced by SPEX.

For example, although Thermanox contains no detectable traces of Ca, V, Cr, Fe, Ni, Cu, or Zn by ICP-MS (Table S3), and exhibits no discernible peaks for first-row transition metals in its X-ray fluorescence spectrum—aside from Fe centered at 6.4 keV (Fig. 2, left panel)—it exhibits the poorest MDLs for Ti through Zn (Table S4). This is primarily due to high levels of inelastic scatter, since the Thermanox cover slip is much thicker than other films. In comparison, silicon nitride, Kapton film, and Ultralene exhibit approximately 100-fold lower inelastic scatter. The strong background in Thermanox renders it a poor substrate for XFM analysis of biological tissue: when brain tissue is deposited on Thermanox, native transition element signals Mn to Zn are difficult to detect (Fig. 2, right panel), unlike the same tissue mounted on Ultralene.

To guide substrate selection for biological XFM, we compared elemental detection performance across commonly used materials. In our evaluation, Ultralene, polypropylene, and Prolene outperformed silicon nitride for Si, P, Cl, Ti, V, Cr, and Mn, while performing comparably for K, Ca, and Fe through Zn. Although all three performed well, Ultralene was selected for further investigation due to its broader use in XFM workflows^50–55^ and the availability of published X-ray dosage data.^53^ Kapton (both film and tape) showed reasonable performance and, given its high thermal stability, was selected for further comparison with Ultralene as a substrate for tandem XFM–LA mapping of tissues. The poorer MDLs for P and Ca in Kapton film and tape are consistent with their gravimetric P and Ca contents (Table S3) and may degrade image quality in biological XFM. Similarly, the poor MDLs for Si in Kapton tape and silicon nitride are expected due to the high Si content in both substrates.

### XFM mapping of biological tissue on Ultralene and Kapton films yields comparable results, except for Ca

Following the determination of XFM minimum detection limits, we next evaluated how Ultralene and Kapton films perform when mapping biological specimens. These two substrates were chosen due to their widespread use, affordability (Table S1) and suitability for depositing cryosectioned tissue. Both can be taped to glass slides for transferring thin cryosections, stored in a freezer and transported on dry ice, allowing for potential long-term storage if beamtime is not readily available. At the beamline, the samples can be moved from the glass slides to a custom-made mount, supporting transferability between XFM and LA-ICP-TOF-MS. During cryosectioning, Ultralene or Kapton films are temporarily taped along their edges to standard glass microscope slides to provide mechanical support for mounting, storage, and transport. At the beamline, the substrate-mounted tissue is not peeled or physically transferred off the film; rather, the tape is gently removed, and the entire film-supported section is lifted from the glass slide and placed into the custom XFM sample mount. These mounts are similar in size to 35 mm projector frames and contain a thin plastic border with an open center, allowing the substrate and tissue to be suspended without distortion during XFM analysis.

**Fig. 3:**
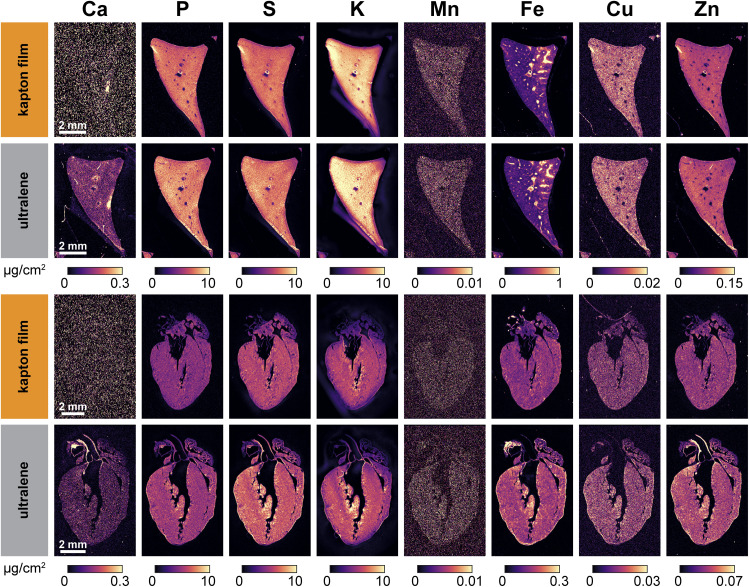
X-ray fluorescence microscopy (XFM) imaging of Ca, P, S, K, Mn, Fe, Cu, and Zn in 20 µm-thick cryosectioned mouse liver (top) and heart (bottom) mounted on either Kapton film (manufactured by SPEX) or Ultralene. Adjacent liver and heart sections were selected and scanned using the same spot size and dwell time to compare Kapton film and Ultralene as XFM substrates. Due to the higher detection limit for Ca on Kapton (see Fig. 1), Ca signals are not discernible in the Kapton-mounted samples but are clearly resolved in those mounted on Ultralene. In contrast, the high endogenous P levels in both tissues overcome the poor limit of detection for P in Kapton film, resulting in clearly visible P maps. For S, K, Mn, Fe, Cu, and Zn, signal quality is comparable between Kapton and Ultralene, consistent with their similar limits of detection (see Fig. 1 and Table S4). The two liver sections were obtained from the same animal at 20 µm apart; the heart sections were collected 460 µm apart from the same animal.

To assess the impact of substrate composition on biological XFM image quality, murine liver and heart were sectioned onto Ultralene and Kapton films and scanned at the 8-BM beamline (Argonne National Laboratory). Comparable elemental maps for P, S, K, Fe, Cu, and Zn were produced on both substrates, which contributed little background interference. However, the resultant Ca maps suffered from high background when samples were mounted on Kapton film, consistent with the high Ca content and poor Ca MDL of the substrate (Table S3 and Fig. 1). In contrast, adjacent tissue sections mounted on Ultralene (Fig. 3) yielded clear Ca maps with minimal background, indicating that Ultralene is the optimal substrate for samples requiring accurate Ca quantification. The low-quality Mn maps are typical of biological XFM—background inelastic scatter competes with the weak Kα fluorescence from trace Mn in biological samples, resulting in noisy images.

### Correlative XFM and LA-ICP-TOF-MS imaging enables complementary quantification of endogenous elements

**Fig. 4:**
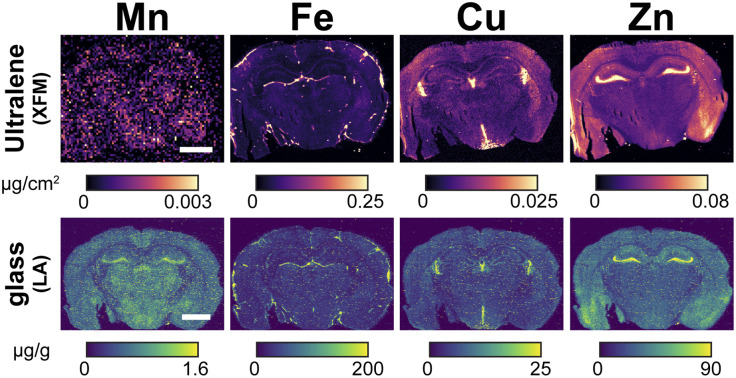
Ultralene and glass are standard substrates for XFM and LA-ICP-TOF-MS, respectively. Murine brain was embedded in OCT media and frozen at −80 °C overnight before cryosectioning. Adjacent 20 µm-thick sections were mounted sequentially onto glass and Ultralene. The glass-mounted section was ablated using LA-ICP-TOF-MS with porcine gelatin calibration standards for quantitation, while the Ultralene-mounted section was analyzed using XFM. Elemental maps of Fe, Cu, and Zn were comparable across both modalities. Notably, the LA-ICP-TOF-MS Mn map exhibited greater clarity due to its higher sensitivity for monoisotopic ^55^Mn, highlighting the complementary strengths of each technique. These results lay the groundwork for future multimodal overlays to evaluate matrix effects and improve quantification of endogenous elements by LA-ICP-TOF-MS. To reduce noise stemming from low Mn fluorescence, the XFM Mn map was smoothed using 4 × 4 pixel binning. Scale bar = 2 mm.

Next, we evaluated whether Ultralene can support multimodal imaging by comparing elemental maps acquired with XFM *versus* LA-ICP-TOF-MS. Two adjacent 20 µm thick cryosectioned murine brain tissues were mounted on Ultralene for XFM analysis or on glass slides for LA-ICP-TOF-MS analysis (Fig. 4). Glass is the substrate of choice for LA-ICP-TOF-MS due to its compatibility with histological techniques such as immunohistochemistry, and because it withstands 266 nm laser ablation with minimal etching or contribution to matrix effects. For these comparisons, we focus on Mn, Fe, Cu, and Zn because they are both biologically informative and quantitatively robust by XFM and LA-ICP-TOF-MS; other elements such as Ca, P, S, and K either lack reliable LA-ICP-TOF-MS calibration under our conditions or yield largely uniform maps and are therefore not shown.

As shown in Fig. 4, elemental maps of Zn, Fe, and Cu are comparable between the two preparations, cementing Ultralene as a suitable substrate for XFM analysis in correlative workflows. In contrast, LA-ICP-TOF-MS provides a notably clearer Mn map, owing to its higher sensitivity and detection of the monoisotopic ^55^Mn isotope. This demonstrates how the two techniques complement each other: XFM offers non-destructive imaging, while LA-ICP-TOF-MS provides enhanced sensitivity for certain elements, together enabling more complete coverage of the tissue metallome. Minor punctate hotspots observed in the LA-ICP-TOF-MS maps arise from small fluctuations in ablation efficiency and aerosol transport rather than true biological enrichments, which is why these features are absent in the XFM images.

While glass is suitable for ablation using a 266 nm nanosecond solid-state laser, the utility of this substrate is laser-platform dependent. Femtosecond systems generate *ca*. 10^3^-fold higher peak power and can fracture glass during tissue ablation.^84^ In addition, shorter-wavelength nanosecond lasers (*e.g.*, 193 nm) have also been reported to ablate or etch certain types of glass substrates under specific fluence conditions.^36^ Substrate ablation performance on other laser platforms thus merits further study.

In these correlative comparisons, XFM and LA-ICP-TOF-MS quantify elemental content in different units (µg cm^−2^*vs.* µg g^−1^), reflecting their distinct physical measurement principles. Converting between these units would require spatially resolved measurements of tissue thickness and density at each pixel; because both parameters vary across cryosections, such a conversion would introduce additional uncertainty rather than improving comparability. For this reason, each modality is presented in its native quantitative units. Although uniform color scales across techniques would be visually attractive, they would imply direct quantitative equivalence between µg cm^−2^ and µg g^−1^. To avoid misleading interpretation, images from each modality are displayed with internally consistent, perceptually uniform color scales appropriate for their respective dynamic ranges.

Furthermore, accurate quantitation by LA-ICP-TOF-MS requires a set of matrix-matched standards. Molten gelatin (10% porcine gelatin spiked with elemental standards) can be pipetted directly onto a pre-cooled cryostat chuck, sectioned at the same thickness as the tissue sample, and mounted onto glass slides.^85^ Since XFM-scanned samples will eventually need to be ablated for correlative analysis, we tested whether substrates compatible with XFM are also suitable for laser ablation using gelatin, a material commonly used to establish LA-ICP-MS calibration curves.

**Fig. 5:**
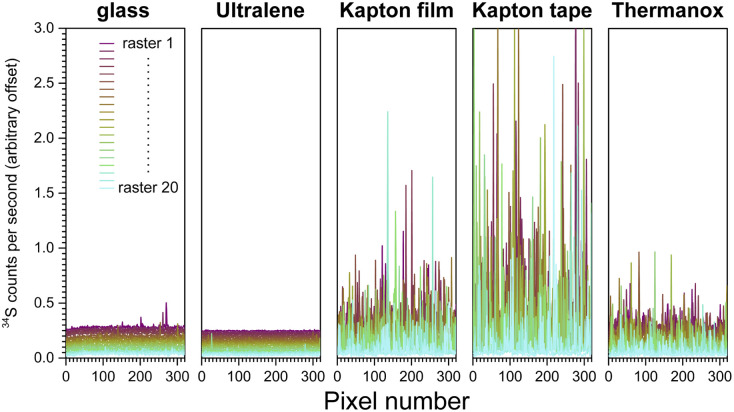
^34^S quantitation by LA-ICP-TOF-MS of 20 µm-thick gelatin sections adhered on glass and various substrates. Each raster is vertically offset by 0.025 units to aid visualization. The stable levels of ^34^S signal across rasters of gelatin on glass and on Ultralene suggest consistent and reliable ablation of the gelatin. The varying levels of ^34^S detected in each raster of gelatin on Kapton film (SPEX), Kapton tape, and Thermanox suggest that gelatin ablates inconsistently on those substrates.

To assess ablation consistency, laser-substrate interactions, and the resultant matrix effects, porcine gelatin was sectioned onto glass, Ultralene, Kapton film, Kapton tape, and Thermanox, and subjected to identical raster scanning conditions. This diagnostic screen was intentionally performed at a single fixed fluence rather than fluence-optimizing for each material, enabling direct comparison of intrinsic substrate-dependent ablation behavior. The ^34^S signal was selected as the metric for ablation consistency because sulfur is endogenous to porcine gelatin, arising primarily from methionine residues,^86^ and is therefore present uniformly across all calibration standards fabricated from the same gelatin source. The icpTOF S2 resolves the major isobaric interferences on ^34^S when data is acquired in KED mode using 5 mL min^−1^ He. We note that differences in gelatin-substrate adhesion also influence ablation behavior: gelatin adheres strongly to Kapton film and tape and can lift in small flakes during ablation, whereas it adheres more weakly to Ultralene. Ultralene yielded the most consistent and clean ablation after glass, while Kapton film and tape performed worst, with Thermanox falling in between (Fig. 5). Because the substrates contain negligible sulfur (Table S3), the observed variation in ^34^S arises from substrate-dependent ablation behavior, including adhesion effects, rather than from substrate co-ablation.

**Fig. 6:**
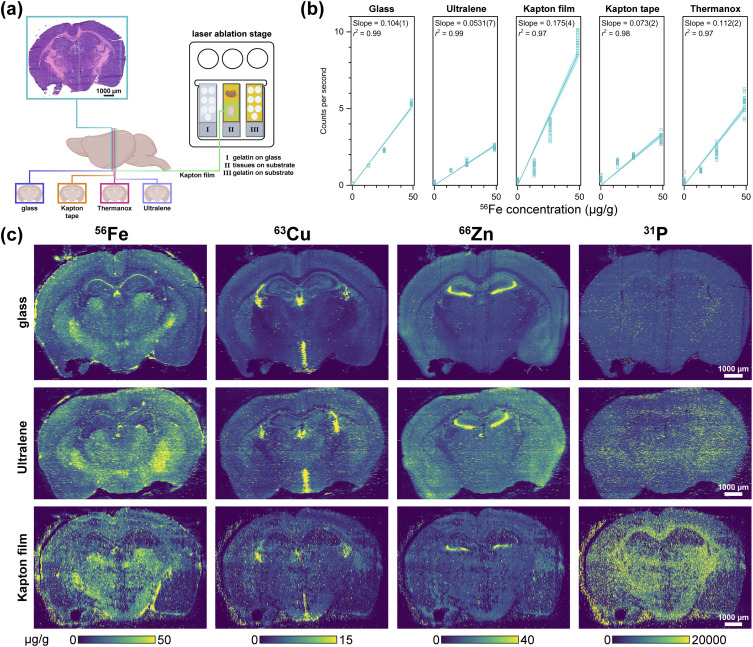
(a) Overview of substrate testing for laser ablation inductively coupled plasma time-of-flight mass spectrometry (LA-ICP-TOF-MS) imaging with biological tissue. Several 20 µm-thick tissue sections from the same organ were collected. One section was used for histochemical characterization, while the remaining sections were mounted on glass or various substrates and scanned to evaluate substrate-induced matrix effects on tissue ablation. In parallel, 20 µm-thick gelatin standards containing varying Fe concentrations were scanned at ten intervals. These gelatin sections were mounted on both glass and substrates to assess substrate-induced matrix effects on the gelatin ablation. (b) Average ^56^Fe counts per second from the ten rasters of gelatin standards containing 0.05, 13.47, 26.29, and 48.58 µg g^−1^ Fe on glass, Ultralene, Kapton film (SPEX), Kapton tape, and Thermanox. The resulting calibrations were fit with a linear regression (blue line) and 95% confidence interval (light blue area). Gelatin on glass and Ultralene showed consistent calibration behavior, while Kapton film, Kapton tape, and Thermanox produced erratic ^56^Fe signals with high variance across replicates. The calibration series shown in panel (b) was generated from a separate set of gelatin standards used to characterize calibration linearity and was not the same set used for tissue quantitation. (c) LA-ICP-TOF-MS elemental maps of mouse brain sections mounted on glass, Ultralene, and Kapton film (SPEX).

Because calibration accuracy can also be influenced by substrate-specific ablation behavior, we next tested how porcine gelatin standards performed when used for elemental quantification during concurrent tissue analysis. To test this, 20 µm-thick murine brain sections were mounted onto XFM-compatible substrates and ablated alongside gelatin standards deposited on the same substrate (Fig. 6a). For example, brain on Ultralene was ablated at the same time as gelatin standards on Ultralene. Glass-mounted gelatin standards were included in every run as a positive control and to monitor ablation efficiency. As expected, the ablation differences observed in Fig. 5 also impacted the substrates' performance as calibration standards (Fig. 6b). While *r*^2^ values for elemental quantification were similar across substrates (see Fig. 6b for Fe; Fig. S2–S6 for ^24^Mg, ^31^P, ^55^Mn, ^63^Cu, and ^66^Zn, respectively), differences in calibration slope and signal intensity demonstrate that standards and tissue samples must be prepared on the same substrate to ensure optimal quantitative accuracy.

### Ultralene and Kapton film outperform other substrates for LA-ICP-TOF-MS mapping

Multimodal mapping and co-registration require images of the same tissue section with aligned dimensions and fiducial markers. Our current multimodal elemental mapping protocol mounts one section onto Ultralene for XFM analysis and the subsequent section on glass for LA-ICP-TOF-MS to compare resultant elemental maps (Fig. 4). However, accurate comparison of endogenous elemental quantitation between the two techniques ideally requires imaging and ablation of the same section using appropriate calibration standards. Therefore, murine brain and kidney tissues were cryosectioned onto XFM-compatible substrates (Ultralene, Kapton film, Kapton tape, and Thermanox) and compared to glass-mounted samples analyzed by LA-ICP-TOF-MS (Fig. 6c and S7). Silicon nitride windows were also examined, but as expected these substrates shattered under a variety of LA imaging conditions and were not further evaluated. Substrate choice significantly influenced elemental maps—kidney cryosections deposited on Ultralene were prone to cracking, while Thermanox introduced the most distortion to elemental contents (Fig. S7). Small punctate hotspots are visible in the LA-ICP-TOF-MS maps and increase from glass to Ultralene to Kapton film; these features arise from minor shot-to-shot variations in ablation efficiency and aerosol transport, which are more pronounced on polymer substrates than on glass, and do not represent true biological enrichments.

Although identical laser dosages were initially applied, it became apparent that the tissue ablation threshold varied by substrate, especially for Kapton film and tape. This observation was consistent with earlier ablation studies using porcine gelatin standards (Fig. 5). To match the quality of glass-based maps, laser power was optimized by ablating a single section at multiple energies. Incremental power increases—1.3, 1.8, 3.8, and 5.7 J cm^−2^ (corresponding to 20%, 30%, 50%, and 60% laser power)—were applied every 100 raster lines to identify optimal laser dosage for each substrate (Fig. S8).

While glass slides offer flat, stable surfaces that promote uniform tissue adhesion free of bubbles or cracks, polymer film substrates proved inherently more difficult to manipulate. Being the thinnest film (Table S1) and prone to static buildup, Ultralene was particularly challenging to secure onto glass slides without introducing wrinkles or changes to the focus plane that degrade quantitation reliability. After taking care to mitigate these artifacts, Ultralene produced elemental maps most comparable to glass, echoing its performance in XFM and supporting its designation as the optimal substrate for multimodal elemental mapping by both XFM and LA-ICP-TOF-MS.

## Conclusions

With the growing interest in multi-omics imaging at the single-cell resolution, identifying substrates compatible with diverse imaging modalities has become increasingly important. For many mass spectrometry imaging (MSI) techniques, including matrix-assisted laser desorption/ionization-MSI (MALDI-MSI), desorption electrospray ionization, and secondary ion mass spectrometry, standard glass slides provide a common, relatively non-destructive substrate. This compatibility enables straightforward sample preparation and facilitates seamless acquisition of multimodal datasets (*e.g.*, proteomics, metabolomics, transcriptomics) from a single tissue section, unlocking previously inaccessible biological insights.^87,88^ Recent studies have combined MALDI with LA-ICP-TOF-MS to layer lipidomics with metallomics, while accounting for the impact of matrix coatings required for MALDI.^89^ These approaches co-register data from the same tissue and yield a more holistic understanding of the complex biological processes.^88,90^

In contrast, combining XFM and LA-ICP-MS presents unique challenges. XFM requires ultrathin, low-background substrates to minimize beam attenuation, while LA-ICP-MS is fully destructive, necessitating careful substrate selection for quantitative elemental mapping. This study systematically evaluated a range of polymer substrates to identify a platform suitable for both techniques and enable sequential multimodal elemental analysis.

Although XFM and LA-ICP-TOF-MS both produce high-resolution, sensitive elemental maps, their combined use offers broader elemental coverage. Among the substrates tested, Ultralene performed best overall, supporting high-quality elemental mapping in both XFM and LA-ICP-TOF-MS. Kapton film showed some utility due to its greater thermal stability but was limited by higher noise in LA-ICP-TOF-MS and elevated Ca background in XFM. Ultralene exhibited low concentrations of interfering elements, MDLs comparable to silicon nitride (the XFM gold standard), and ablation performance on par with glass (the LA-ICP-TOF-MS gold standard).

In sum, Ultralene emerges as a practical and effective common substrate for multimodal elemental imaging by XFM and LA-ICP-TOF-MS. Its dual compatibility facilitates same-section analysis, helps mitigate matrix effects, and offers a cost-effective alternative to fragile or high-background substrates. This work provides a foundation for integrated, quantitative metallomic workflows that will enhance our understanding of elemental biology across tissues and disease models.

## Author contributions

D. Z. Z., K. W. M. and T. V. O. conceptualized the ideas of the manuscript. A. M. C. analysed the XFM dataset. Q. J. prepared and ran the samples on the XFM. N. S. and S. H. A. prepared the murine samples for LA-ICP-TOF-MS analysis. D. Z. Z., K. W. M., S. H. A., A. M. C., and T. V. O wrote the manuscript. All figures were generated by D. Z. Z., with contributions from S. H. A. and A. M. C.

## Conflicts of interest

There are no conflicts to declare.

## Supplementary Material

JA-041-D5JA00371G-s001

## Data Availability

The data supporting the findings of this study are available within the article and its supplementary information (SI). Other relevant data can be shared upon reasonable request to the authors. Supplementary information: SI tables of commercial substrates, experimental parameters, elemental contents and minimum detection limits, SI figures of calibration curves and laser ablation maps. See DOI: https://doi.org/10.1039/d5ja00371g.
