# Supplementary material for: Substrate and standard evaluation for correlative elemental mapping of biological samples by X-ray fluorescence microscopy and laser ablation ICP-MS
Source: J Anal At Spectrom. 2026 Jan 16;41(2):708–21. doi: 10.1039/d5ja00371g (PMC12809665; doi:10.1039/d5ja00371g)
Supplement: JA-041-D5JA00371G-s001 [file JA-041-D5JA00371G-s001.pdf]

Supplementary Information (SI) for Journal of Analytical Atomic Spectrometry.
This journal is © The Royal Society of Chemistry 2026

Electronic Supplementary Information for:

Substrate and standard evaluation for correlative elemental mapping of biological samples by X-ray fluorescence microscopy and laser ablation ICP-MS

David Z. Zee,[1,2] Soo Hyun Ahn,[1] Andrew M. Crawford,[1,2]
Niharika Sinha,[1,2] Qiaoling Jin,[3,4] Chris Jacobsen,[4] Evan Maxey,[3]
Barry Lai,[3] Keith W. MacRenaris,*[1,2] and Thomas V. O'Halloran*[1,2]

[1]The Elemental Health Institute and the Departments of Microbiology, Genetics, and Immunology and of [2]Chemistry, Michigan State University, East Lansing, Michigan 48824, United States.

[3]X-ray Science Division, Advanced Photon Source, Argonne National Laboratory, Lemont, Illinois 60439, United States

[4]Department of Physics and Astronomy, Northwestern University, Evanston, Illinois 60208, United States

*Email: macrenar@msu.edu; ohallor8@msu.edu

J. Anal. At. Spectrom.

Table of Contents

Table S1. Commercial substrate materials examined in this study. **S3**

Table S2. Experimental parameters for LA-ICP-TOF-MS. **S4**

Figure S1. Chemical structures of the substrates with well-characterized compositions. **S5**

Table S3. Elemental contents of substrates used as determined by ICP-MS analysis of acid digests. **S6**

Table S4. Elemental minimum detection limits of X-ray fluorescence microscopy when using a given substrate. **S7**

Figure S2–S6. LA-ICP-TOF-MS calibration curves for gelatin standards on various substrates. **S8–S12**

Figure S7. LA-ICP-TOF-MS maps of murine brain and kidney on substrates. **S13**

Figure S8. Laser fluence testing of murine brain on substrates. **S14**

Table S1. Commercial substrate materials examined in this study.

name	supplier	catalog no.	lot no.	thickness (µm)	cost per area[c] ($ / cm^2)
polypropylene	Chemplex	3020	820618S1A	6.0	0.009
Prolene	Chemplex	3018A	840427S1A	3.0	0.01
Mylar	Chemplex	3012	830121S1A	2.5	0.007
Ultra-Polyester	Chemplex	3090	820627S1A	1.5	0.01
Etnom	Chemplex	3095	820910S1A	1.5	0.01
Kapton	Chemplex	3022-5	–[a]	7.5	0.04
Ultralene	SPEX / Cole-Parmer	3525	–[a]	4.0	0.003
Kapton	SPEX / Cole-Parmer	3511	–[a]	8.0	0.01
Zythene	Chemplex	3081	110477S1A	6.0	0.01
Thermanox	Nunc / ThermoFisher	150067	–[a]	200	14
silicon nitride	Norcada	NX151000D	–[a]	0.200	190
Kapton tape	Electron Microscopy Sciences	77708-04	–[a]	68.6[b]	0.01

[a]Information not provided.
[b]Total thickness comprising a 25.4 µm thick layer of Kapton HN and a 43.2 µm thick layer of silicone adhesive.
[c]Estimated using retail prices as of April 2025, not including any discounts.

Table S2. Experimental parameters for LA-ICP-TOF-MS.

ICP-MS parameters (Tofwerk S2)	value
RF power	1550 W
sampling depth	4.9 mm
cone material	nickel
cone insert	3.5 mm
plasma gas flow	14.0 L/min
auxillary gas flow	8.0 L/min
nebulizer gas flow	0.95–1.01 L/min
measurement mode	CCT mode
CCT gas flow (100% He)	5 mL/min
CCT focus lens	−19.5 V
CCT entry lens	−180 V
CCT mass	250 V
CCT bias	10 V
CCT exit lens	−200 V
**time-of-flight parameters (Tofwerk S2)**	**value**
m/*z* range	14−256
resolution, $m/\Delta m$	1000
ODG settings	24 ms
notch bias	−80 V
notch (40 amu)	1.6 V
notch (36 amu)	1 V
notch (28 amu)	2 V
notch (15.8 amu)	2 V
**laser ablation parameters (ESL imageBIO266)**	**value**
spot size	10 µm
interline distance (*y*)	10 µm
overlap (*x*)	0 µm
repetition rate	100 Hz
laser power	60%
laser fluence	15 J/cm^2
sample energy	0.010–0.015 mJ
imaging cup flow rate (He)	200 mL/min
imaging chamber flow rate (He)	250 mL/min
PEEK Tubing I.D.	0.75 mm

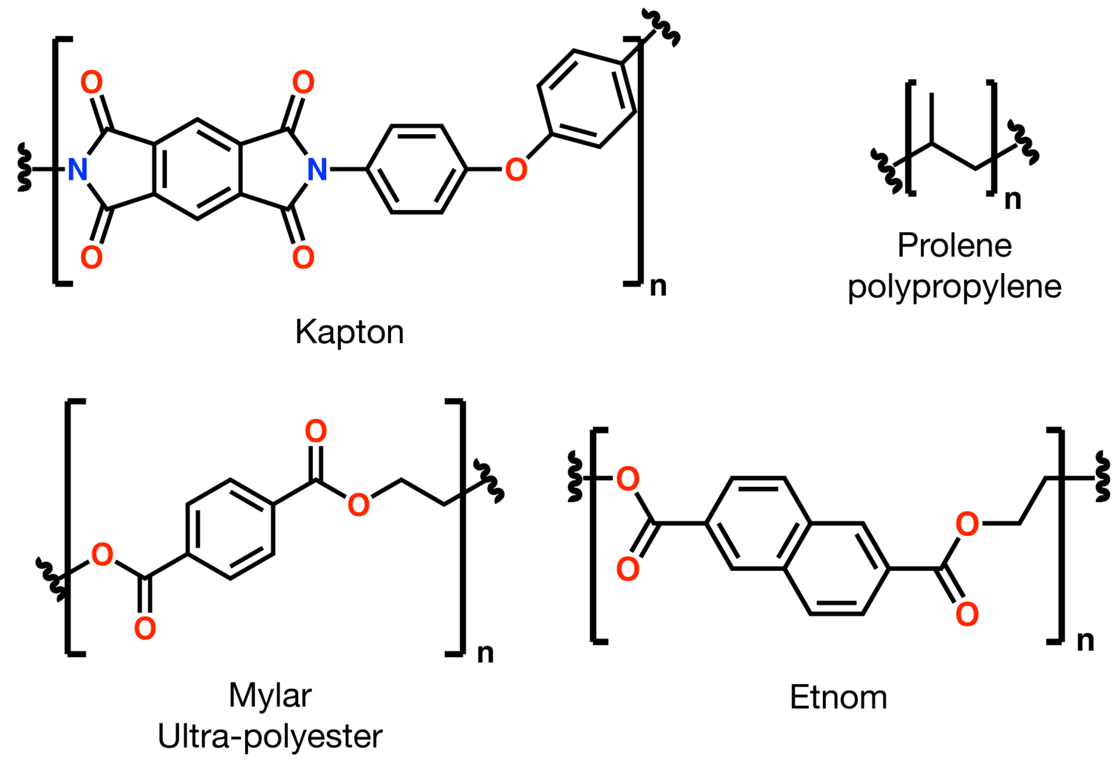

Figure S1. Chemical structures of the substrates with well-characterized compositions.

Table S3. Elemental contents of substrates as determined by ICP-MS analysis of acid digests.[a]

	elemental content (µg element / g substrate)									
	Ultralene (n = 2)	Kapton film[b] (n = 2)	Kapton film[c] (n = 2)	polypropylene (n = 2)	Prolene (n = 2)	Mylar (n = 2)	Ultra-polyester (n = 2)	Etnom (n = 2)	Zythene (n = 2)	Thermanox (n = 4)
Mg	–[d]	–[d]	–[d]	–[d]	–[d]	–[d]	–[d]	–[d]	–[d]	67.3
P	–[d]	238	385	–[d]	–[d]	1,130	–[d]	–[d]	–[d]	43.5
S	–[d]	–[d]	–[d]	–[d]	–[d]	–[d]	–[d]	–[d]	–[d]	–[d]
Ca	144	320	546	–[d]	–[d]	2,060	2,220	204	96.2	–[d]
V	–[d]	–[d]	–[d]	–[d]	–[d]	–[d]	–[d]	–[d]	–[d]	–[d]
Cr	–[d]	–[d]	–[d]	–[d]	–[d]	–[d]	–[d]	–[d]	–[d]	–[d]
Mn	–[d]	–[d]	–[d]	–[d]	–[d]	–[d]	–[d]	59.2	58.9	21.8
Fe	–[d]	–[d]	–[d]	–[d]	–[d]	–[d]	–[d]	–[d]	–[d]	–[d]
Co	–[d]	–[d]	–[d]	–[d]	–[d]	–[d]	–[d]	–[d]	–[d]	0.669
Ni	–[d]	–[d]	–[d]	–[d]	–[d]	–[d]	–[d]	–[d]	–[d]	–[d]
Cu	–[d]	–[d]	–[d]	–[d]	–[d]	–[d]	–[d]	–[d]	–[d]	–[d]
Zn	–[d]	–[d]	–[d]	–[d]	–[d]	–[d]	–[d]	–[d]	–[d]	–[d]
Se	–[d]	–[d]	–[d]	–[d]	–[d]	–[d]	–[d]	–[d]	–[d]	–[d]
Mo	–[d]	–[d]	–[d]	–[d]	–[d]	–[d]	–[d]	–[d]	–[d]	–[d]

[a]Elemental content in substrates reported as an average of n experimental replicates. [b]Kapton film manufactured by SPEX. [c]Kapton film manufactured by Chemplex. [d]Result was less than that of the method blank (240 µL of acid digest diluted to 10 mL—Mg: 1.466; P: 7.227; S: 17.486; Ca: 9.988, V: 0.029, Cr: 0.186; Mn: 0.062; Fe: 1.513; Co: 0.003; Ni: 0.498; Cu: 13.628; Zn: 0.582; Se: 0.004; Mo: 0.007 ng element / g ICP sample).

Table S4. Elemental minimum detection limits X-ray fluorescence microscopy when using a given substrate.[a]

	XFM minimum detection limit with a given substrate ($\mu g\ cm^{-2}$)[b]											
	silicon nitride	Ultralene	Kapton film[c]	Kapton film[d]	polypropylene	Prolene	Mylar	Ultra-polyester	Etnom	Zythene	Thermanox	Kapton tape
Si	6.826	0.3142	0.5589	0.5900	0.3510	0.3389	0.5293	0.4185	0.9582	1.383	1.549	9.250
P	0.3228	0.2602	1.037	1.114	0.3074	0.2867	1.194	0.5059	0.3244	0.4553	0.8964	1.649
S	0.09515	0.1907	0.3031	0.3314	0.2321	0.2296	0.2818	0.2710	0.1876	0.2128	0.5539	0.7098
Cl	0.2458	0.08375	0.1226	0.1338	0.1014	0.09749	0.1115	0.1072	0.07938	0.09068	0.3835	0.3017
K	0.03957	0.04744	0.06443	0.07757	0.06276	0.06236	0.07444	0.07326	0.04910	0.05271	0.2054	0.1763
Ca	0.02111	0.03381	0.9654	0.6879	0.04161	0.03372	0.5784	0.3636	0.06598	0.07577	0.4748	0.1612
Ti	0.01374	0.01085	0.01717	0.01764	0.01334	0.01278	0.04012	0.02127	9.842×10^{-3}	0.01446	0.09650	0.05815
V	7.102×10^{-3}	6.256×10^{-3}	0.01161	0.01223	7.934×10^{-3}	5.387×10^{-3}	0.01338	7.995×10^{-3}	6.496×10^{-3}	9.290×10^{-3}	0.06027	0.04250
Cr	9.000×10^{-3}	6.700×10^{-3}	9.774×10^{-3}	0.01069	6.960×10^{-3}	6.279×10^{-3}	6.104×10^{-3}	6.384×10^{-3}	5.742×10^{-3}	8.504×10^{-3}	0.08699	0.03349
Mn	0.01931	5.937×10^{-3}	7.952×10^{-3}	8.210×10^{-3}	6.055×10^{-3}	5.759×10^{-3}	6.440×10^{-3}	6.759×10^{-3}	0.02106	0.03524	0.1032	0.02615
Fe	8.902×10^{-3}	9.654×10^{-3}	1.312×10^{-3}	0.01487	0.01016	0.01010	1.249×10^{-3}	0.01119	0.01179	0.01679	0.05196	0.03215
Co	3.465×10^{-3}	3.583×10^{-3}	5.442×10^{-3}	5.601×10^{-3}	3.747×10^{-3}	3.344×10^{-3}	4.065×10^{-3}	3.946×10^{-3}	2.991×10^{-3}	4.080×10^{-3}	0.03099	0.01712
Ni	2.634×10^{-3}	4.772×10^{-3}	7.207×10^{-3}	7.333×10^{-3}	4.970×10^{-3}	4.487×10^{-3}	4.917×10^{-3}	5.016×10^{-3}	3.856×10^{-3}	5.453×10^{-3}	0.02723	0.02221
Cu	5.629×10^{-3}	6.532×10^{-3}	7.173×10^{-3}	7.841×10^{-3}	6.590×10^{-3}	6.583×10^{-3}	7.080×10^{-3}	7.838×10^{-3}	6.470×10^{-3}	6.853×10^{-3}	0.03475	0.01614
Zn	3.488×10^{-3}	4.114×10^{-3}	5.856×10^{-3}	5.729×10^{-3}	4.019×10^{-3}	3.818×10^{-3}	4.106×10^{-3}	4.038×10^{-3}	3.510×10^{-3}	4.176×10^{-3}	0.04045	0.01619

[a]As determined with X-ray fluorescence microscopy at 8BM-B (Advanced Photon Source, Argonne National Laboratory) with dwell time = 0.050 s and incident energy of 10 keV. [b]Minimum detection limit = 3σ, where σ is the standard deviation of areal elemental content across all pixels. [c]Kapton film manufactured by SPEX. [d]Kapton film manufactured by Chemplex

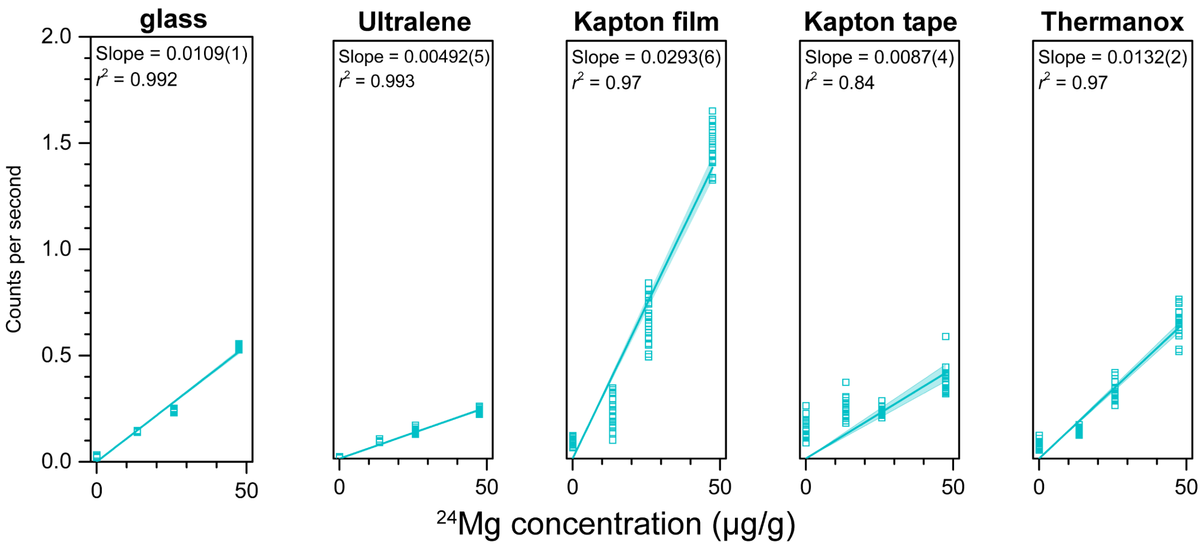

Figure S2. Calibration curves for ^{24}Mg quantitation by LA-ICP-TOF-MS of gelatin standards adhered on substrates. The Kapton film was manufactered by SPEX.

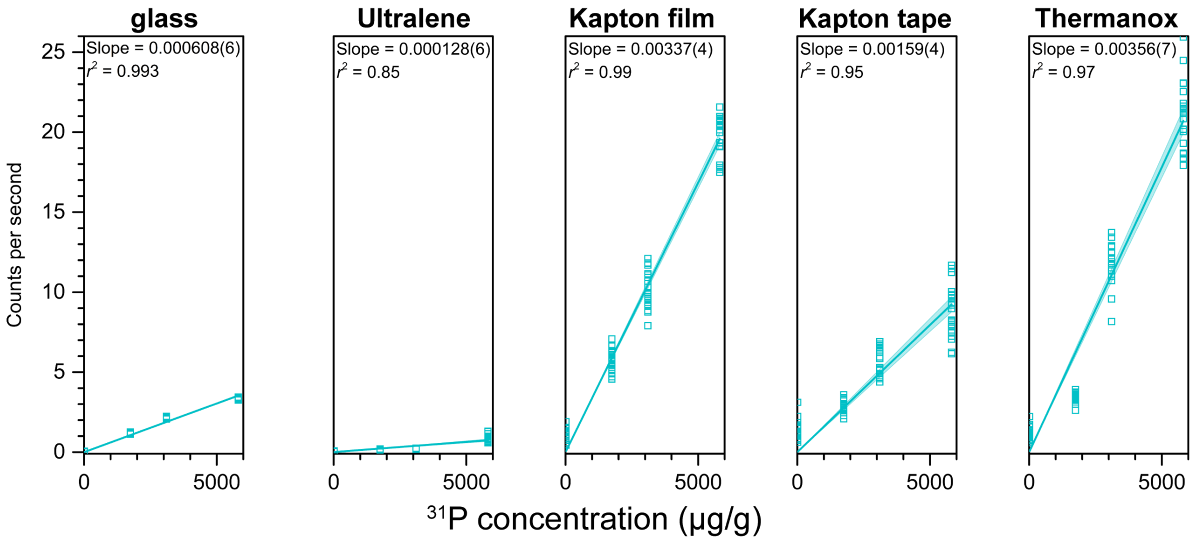

Figure S3. Calibration curves for ^{31}P quantitation by LA-ICP-TOF-MS of gelatin standards adhered on substrates. The Kapton film was manufactered by SPEX.

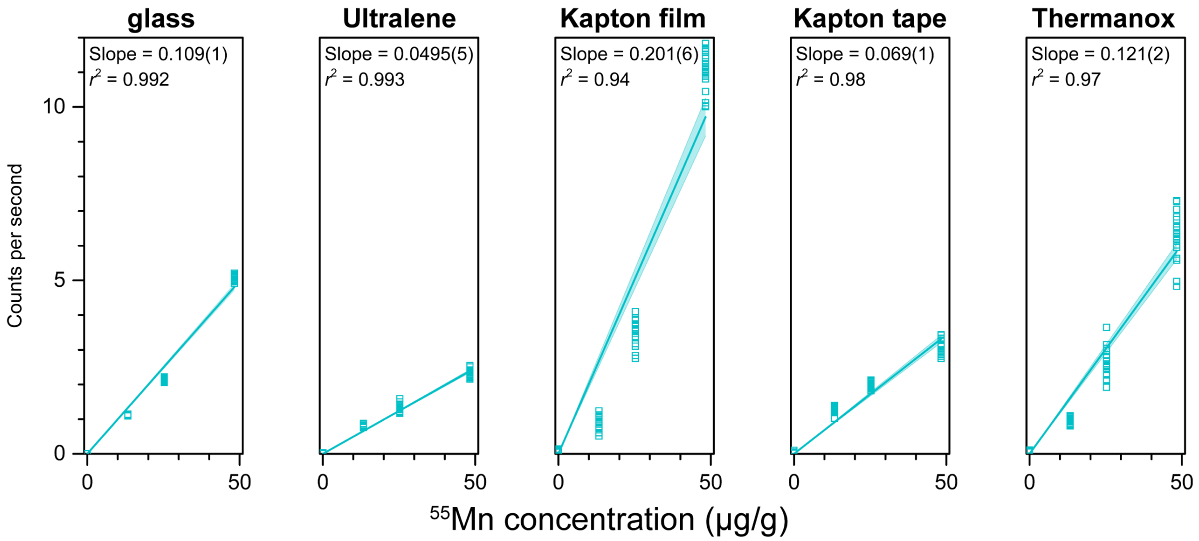

Figure S4. Calibration curves for ^{55}Mn quantitation by LA-ICP-TOF-MS of gelatin standards adhered on substrates. The Kapton film was manufactered by SPEX.

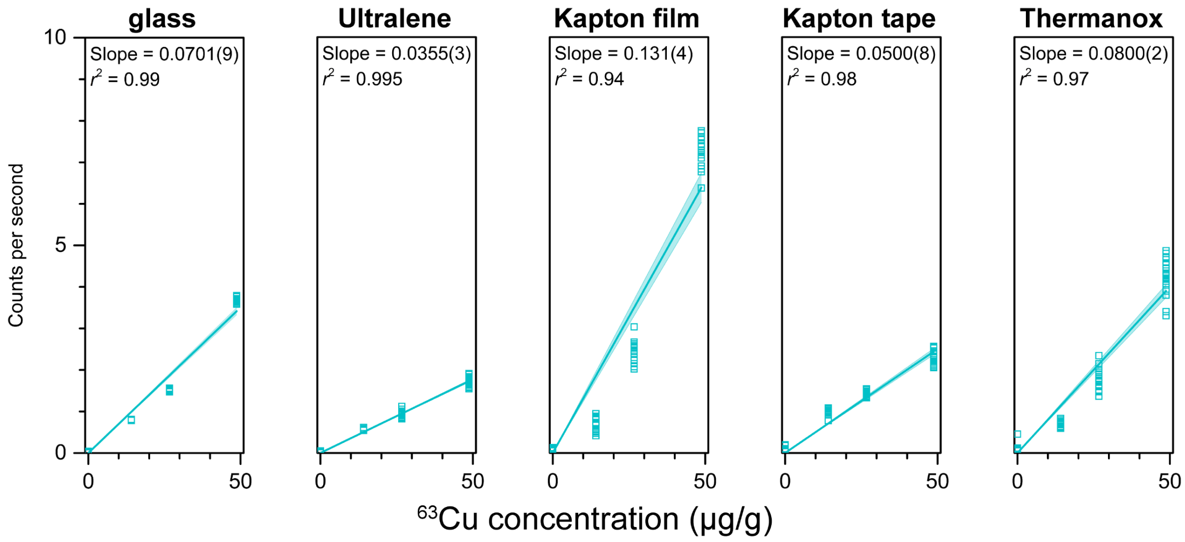

Figure S5. Calibration curves for ^{63}Cu quantitation by LA-ICP-TOF-MS of gelatin standards adhered on substrates. The Kapton film was manufactered by SPEX.

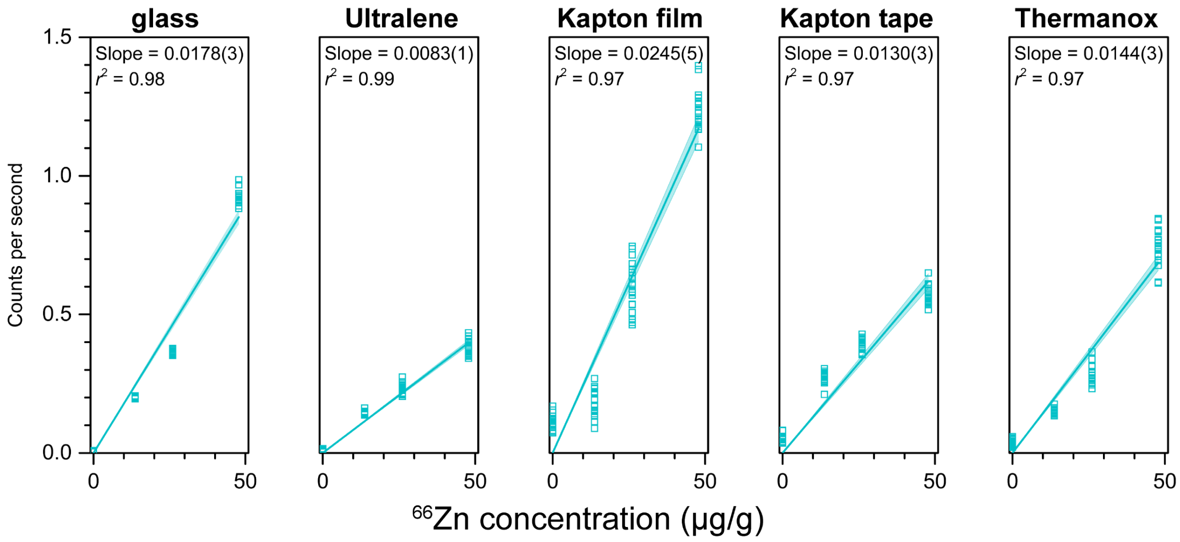

Figure S6. Calibration curves for ^{66}Zn quantitation by LA-ICP-TOF-MS of gelatin standards adhered on substrates. The Kapton film was manufactered by SPEX.

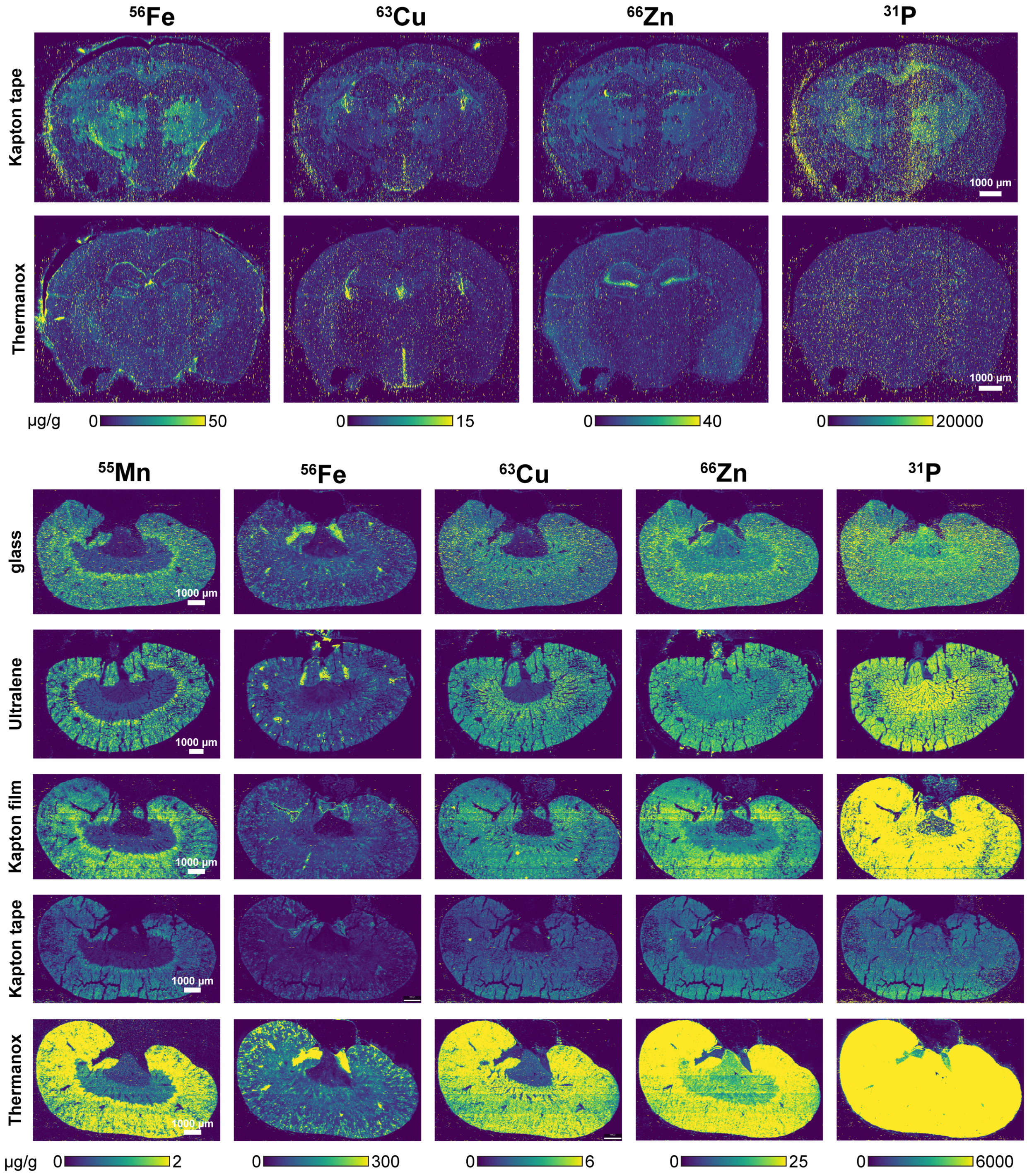

Figure S7. Elemental maps of ^{56}Fe, ^{63}Cu, ^{66}Zn, ^{31}P of mouse brain on Kapton tape, and Thermanox and elemental maps of ^{55}Mn, ^{56}Fe, ^{63}Cu, ^{66}Zn, and ^{31}P of mouse kidney on glass, Ultralene, Kapton film (SPEX), Kapton tape, and Thermanox show substrate-dependent ablation characterstics. Mouse brain was embedded in OCT media and frozen at −80 °C freezer overnight. Mouse kidney was embedded in OCT media and was frozen by plunging the plastic mold into isopentane chilled over liquid nitrogen. The tissues were sectioned at 20 µm thickness sequentially onto the substrates. All samples were ablated at laser fluence of 2.7 J/cm^2 (corresponding to 40% laser power) with 10-µm spot size. Brain scans on glass, Ultralene, and Kapton film are presented on the main manuscript (see Fig. 6).

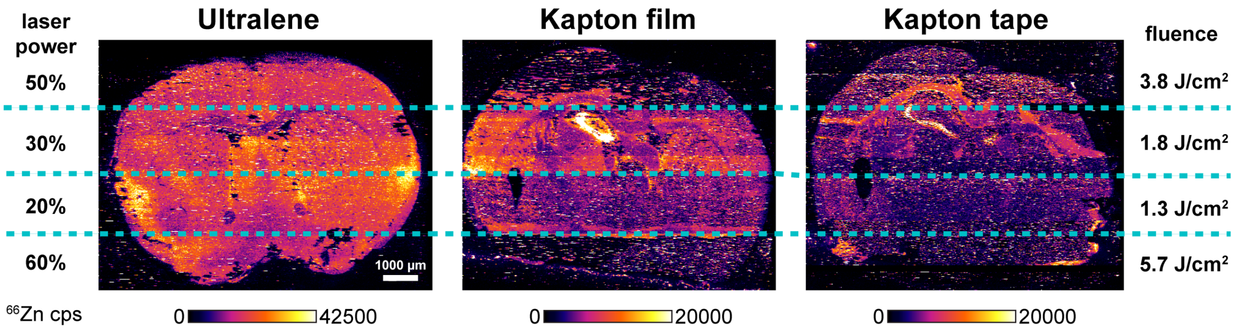

Figure S8. Laser fluence testing ("Easter Egg" power test) of 20-µm thick murine brain tissue deposited onto Ultralene, Kapton film (SPEX), and Kapton tape to determine optimal ablation conditions for each substrate. Each panel shows four horizontal raster regions corresponding to fluences of 3.8, 1.8, 1.3, and 5.7 J/cm^2 (50%, 30%, 20% and 60% laser power, top to bottom). Maps display raw ^{66}Zn counts per second (cps). Horizontal guides demarcate raster regions to facilitate comparison across fluence settings. Lower fluence produced cleaner ablation for Kapton film and Kapton tape, while Ultralene supported a wider fluence range.
